# Identification of distinct physical activity profiles through adolescence: a longitudinal qualitative description study

**DOI:** 10.3389/fspor.2024.1230999

**Published:** 2024-08-22

**Authors:** Mathieu Bélanger, Julie Goguen, Jacinthe Beauchamp, François Gallant, Anika Boucher, Jean-Sébastien Chevarie, Sara DeGrâce, Yanis Saheb, Maryse Gagnon, Isabelle Doré, Catherine M. Sabiston

**Affiliations:** ^1^ Department of Family and Emergency Medicine, Université de Sherbrooke, Sherbrooke, QC, Canada; ^2^ Centre de Formation Médicale du Nouveau-Brunswick, Moncton, NB, Canada; ^3^ Office of Research Services, Vitalité Health Network, Moncton, NB, Canada; ^4^ Faculty of Medicine and Health Sciences, Université de Sherbrooke, Sherbrooke, QC, Canada; ^5^ School of Kinesiology and Physical Activity Sciences, Faculty of Medicine, Université de Montréal, Montréal, QC, Canada; ^6^ Department of Social and Preventive Medicine, École de Santé Publique, Université de Montréal, Montréal, QC, Canada; ^7^ Faculty of Kinesiology and Physical Education, University of Toronto, Toronto, ON, Canada

**Keywords:** physical activity profiles, adolescence, longitudinal qualitative description, sport participation, motive for physical activity

## Abstract

**Introduction:**

We aimed to better understand longitudinal physical activity experiences among initially active adolescents and to identify and describe distinct physical activity profiles.

**Methods:**

A sample of 23 physically active participants [52% female; mean age = 12.2 (0.6) years at study inception] were selected from the MATCH study to take part in this nested qualitative descriptive study. Participants were interviewed once a year for six years. Following individual-level analyses, profiles were identified based on similarity of longitudinal experiences.

**Results:**

Four profiles captured participants' experiences: Independents (those who progressively seek activities that cater to their pursuit of autonomy); Multitaskers (those who participate in many different sports as an integral part of their lifestyle); Specialists (those who are dedicated to becoming the best they can be at one sport); Undecided (those who take part in physical activity to occupy time).

**Discussion:**

The exploration of longitudinal physical activity experiences led to the identification of distinct profiles that could be targets for tailored interventions, theory development, and participation models.

## Introduction

Over 80% of adolescents globally are insufficiently physically active (1). However, those who succeed in remaining physically active during adolescence have a higher likelihood of becoming physically active adults (2–6) and avoiding mental health challenges (7, 8), cardiovascular diseases (9–11), cancer (12, 13) and other chronic conditions (14). Learning from the experiences of individuals who sustain participation in physical activity (PA) throughout adolescence could help inform the design of strategies to support optimal PA levels during this important period of growth and development. However, most research aiming to describe experiences with PA during adolescence is cross-sectional, does not differentiate between sub-groups of youth with different PA experiences, and focuses on individuals with low levels of PA (15, 16). To date, the only studies focusing on physically active adolescents are devoted to describing their experience in a specific sport, which limits generalisability of results (17). Despite their limitations, these previous studies suggest that different groups of individuals can maintain or discontinue participation in sports or other physical activities for different reasons. For example, in their review of qualitative studies assessing transitions of athletes from development programs to highly competitive sport participation, Drew et al. (17) highlighted the presence of considerable inter-individual variability in approaches and perceptions related to change in sport participation. Previous studies among young athletes also suggest that maintenance of sport participation can revolve around performance for some, whereas enjoyment and maintaining friendships are fundamental to others (18). Differences among young athletes were also identified with regards to the place sports take in their lives relative to other facets of life (19). Specifically, whereas some young athletes place great importance on sport, others prioritize the demands of academic tasks (20, 21). Qualitative studies on general PA participation also suggest different individuals may perceive different benefits from participation in PA, even among participants within the same PA (22, 23). Although common external factors may be responsible for differences in the perceived quality of experience and likelihood of maintaining participation in PA (24, 25), it is likely that within-individual characteristics also contribute to explaining how and why different groups of youth engage in and experience various types of physical activities differently throughout adolescence.

In addition to previous studies, theoretical perspectives, including self-determination theory (SDT), can help develop ideas as to how different factors can influence the emergence of different profiles of PA throughout adolescence. SDT is founded on the notion that individuals behave according to an interaction between extrinsic forces, intrinsic motivations and basic psychological needs (26). As children transition into adolescence, many factors, such as increasing levels of competitiveness in sports (27, 28), changes in goals (29, 30), perceived parental support (31–33), and competing interests (34) can either support or thwart adolescents’ needs for competence, autonomy, and relatedness, thus impacting their motivation for, and level of participation in, PA. Furthermore, previous studies suggest that these needs are differentially associated with different types of PA: the need for competence predicts participation in an organised exercise program (35), relatedness relates to maintenance of sport participation (36), and autonomy is associated with a general measure of PA (37). Different motives may also have distinct associations with sustainment of participation in specific physical activities. For example, strength conditioning activities are associated with fitness-related motives, participation in aerobics with appearance motives, and dancing with enjoyment as well as social and competence motives (38).

In summary, although we need to understand the longitudinal PA experience of physically active adolescents, previous longitudinal qualitative research on PA has limited its scope to elite sport participation or participation in specific sports (21, 39–41). Furthermore, other qualitative studies could only offer superficial understanding of adolescent PA as they were exclusively cross-sectional and did not differentiate across individuals with distinct experiences with PA (16). Nevertheless, results from previous studies suggest that it is possible for elements of theories, including SDT, to contribute to the underlying experience surrounding PA participation. The literature also suggests that differences in motives for participation may explain the existence of different PA profiles (38). Therefore, the purpose of this study was to identify sub-groups of adolescents with different patterns of experience with PA and to provide a description of the experiences and motives of initially active adolescents in these PA profiles over six years.

## Methods

Qualitative description is well suited for inquiries focused on defining and gaining insights into poorly understood phenomena (42–45), such as “longitudinal experiences of PA” in the context of adolescence, to inform future programs and practices. Consistent with a qualitative description study, a primarily inductive methodology was adopted, which acknowledges the subjectivity of experiences of both the participants and the researchers. Participants' perspectives, experiences, and descriptions largely directed the interviews used for data collection and researchers' discussions during the analyses (46). Furthermore, the researchers define their own epistemological and ontological position as that of subjectivist relativism, with the understanding that reality varies across individuals, whereby adolescents experience and perceive their own realities, interpretations, and meanings that help to understand the phenomenon (43, 45, 46). This qualitative description therefore embraces a malleable commitment to theory and guiding frameworks (46).

### Participants

This longitudinal qualitative description study is based on a subsample of 23 participants purposefully drawn from the MATCH study, an ongoing prospective study. Specifically, 929 students from 17 schools across the REGION, COUNTRY were recruited. Quantitative questionnaires were first administered in Fall 2011, when students were in Grades 5 or 6 (10–12 years old), and were repeated approximately every four months (24 survey cycles) throughout subsequent school years. Schools included a combination of French- and English-speaking students from high, moderate, and low socioeconomic status neighbourhoods located in rural and urban areas. In the questionnaire, participants self-reported their physical activity level using a 2-item questionnaire (47) and their involvement in 36 different physical activities using a checklist which also questioned the context in which participation took place.

The qualitative component started in Spring 2013 (year 2 of MATCH) and involved annual data collection over six years with a goal to provide a deeper understanding of the lived experiences of youth. Specifically, since quantitative studies have limitations in their capacity to capture feelings, emotions, and perceptions of individuals in relation to their experience with PA, the qualitative component of the study aimed to fulfil this gap. Moreover, given the interest in understanding the particularities that contribute to sustaining (or not) participation in PA, this qualitative component focused on individuals who were physically active at the beginning of adolescence. Specifically, students were selected among the most physically active participants, as determined from responses in the first four cycles of quantitative data collection (i.e., >1 year of data). Six students were purposely recruited to represent participants with their main PA involvement in either one of the following types of PA: (a) team-based; (b) individual; (c) organised; or (d) non-organised physical activities. There was no intent to study participants according to these types of PA. Rather, this sampling strategy aimed to ensure participants represented a wide variety of PA types and experiences but overlap among the types of physical activities practiced by participants was expected. The purposive convenience sampling approach also intentionally identified equal numbers of boys and girls within each category. All 24 participants invited to take part in the qualitative component of the study accepted the invitation and their parents consented. One participant was excluded from further qualitative data collection after the first interview since interview data suggested this participant was not physically active and the goal of the qualitative study was to follow-up participants who were physically active at the beginning of adolescence. All other participants were retained, regardless of their participation in PA after the first interview. As such, the final sample was 23 adolescents, including 12 girls and 11 boys who had a mean age of 12.2 (standard deviation, 0.6) years at their first interview. MATCH received ethics approval from Université de Sherbrooke Institutional Ethics Review Board. All participants understood the study objectives and provided assent to participate in this study; consent was provided by their parents. Throughout the manuscript, “physical activity (PA)” is used to define any leisure-based activity that involves body movements, while “sport” more specifically refers to any PA performed according to a set of rules individually or as part of a team where participants pursue a defined goal (48, 49).

### Data collection

Individual face-to-face interviews were conducted once per year, for a total of six interviews per participant. This avoided retrospective recalls and enabled timely insights into changes as each participant aged. The interviews took place in a private room in the participants' schools, lasted between 25 and 90 min, were audio-recorded and transcribed verbatim. Three authors (MB, JG, JB) conducted 75% of the interviews while research assistants (*n* = 5) conducted the remaining interviews. Before every data collection cycle, interviewers gathered to review the interview guide and to practice simulation scenarios in order to develop a shared understanding of the questions and to establish an approach to the interviews that was standardized.

### Interview guide

The interview guide (Supplementary Appendix 1) was minimally semi-structured and included broad questions aimed at exploring experiences of PA (e.g., “*What is it like to do this activity?*”). Although the approach was primarily inductive, we used existing theories to guide data collection. Specifically, interviews drew from SDT by seeking to understand participants’ reasons for participation in PA (e.g., “*What does doing activity X bring you? What makes you want to continue?*”), as well as perceptions of competence (e.g., “*If you think about knowledge, abilities and skills in relation to activity X, how would you describe yourself?*”), relatedness (e.g., “*Tell me about how you interact with others when you do activity X*”), and autonomy (e.g., “*Who decided you would participate in activity X?*”). After baseline interviews, the evolution of the experiences and circumstances impacting participants were also explored (e.g., “*What is it like compared to last year?*”). All interview questions and responses were facilitated with validation and clarification probes (e.g., “*How do you feel about that? What do you mean by…?*”).

### Data analysis

To foster data-driven theorizing and allow previously overlooked issues in the literature to be uncovered, we started with an inductive approach with the primary intention of identifying emergent themes inductively, rather than making inferences. Themes were grouped together into higher-order themes based on commonalities (50). Themes served to understand participants’ experiences, as well as create a narrative of each participants' PA trajectory to help determine the nature of any change, and facilitate recognition of influential factors or triggers in follow up interviews (51). To this end, one author read all interview transcripts from one participant in their chronological order to create a participant-specific narrative, inductively noting the characteristics of the participant's distinctive longitudinal experiences. Aligning with a data analysis spiral (52), the author then re-read all transcripts while coding them using the ATLAS.ti software (version 9.1.7 for Windows) and completing the participant's narrative (46). Six authors (JB, AB, JSC, SD, YS, MG), all bilingual, independently went through this process, each with three to five participants. Two other authors (MB, JG), who had collected most of the data, each read the full transcripts of half of the participants and developed their own participant narratives to verify consistency of coding over time and across coders. Finally, to further establish credibility, three other authors (FG, ID, CMS), who did not participate in the previous steps, served as peer examiners throughout the study by scrutinizing and iteratively challenging the analytical process and the interpretation of findings.

During the analysis process, the authors held several meetings to discuss overall understanding of each participant's experience (i.e., narrative) as well as their coding of key elements of the interviews. This collaborative rigor helped the authors develop a shared understanding of both the data and the experience of participants (53). The basis for creating profiles was established by presenting and discussing characteristics of the longitudinal PA trajectory of each participant (54). Participants with similar experiences and attributes were grouped together, resulting in the creation of profiles through within-group collective interpretative accounts of convergent and divergent longitudinal experiences (55). The authors then finalized the analysis by supplementing the inductive analysis with deductive identification of how each of the profiles and themes aligned or contrasted with theoretical positions. Quotes were selected to illustrate the characteristics and themes. Data from Francophone participants (*n* = 16) were translated into English at the time of manuscript writing. To maintain anonymity, participants' identifiers were replaced by pseudonyms and the name of the PA that they practiced was removed.

Finally, we present participants' PA levels over the first 24 cycles of quantitative data collection. This is provided as complementary descriptive information but was not used to inform the development of the PA profiles described herein. Estimates of PA levels are derived from a two-item questionnaire where participants reported the number of days during which they engaged in moderate to vigorous PA for at least 60 min, both in the previous week and in a typical week (47).

## Results

**Figure 1:**
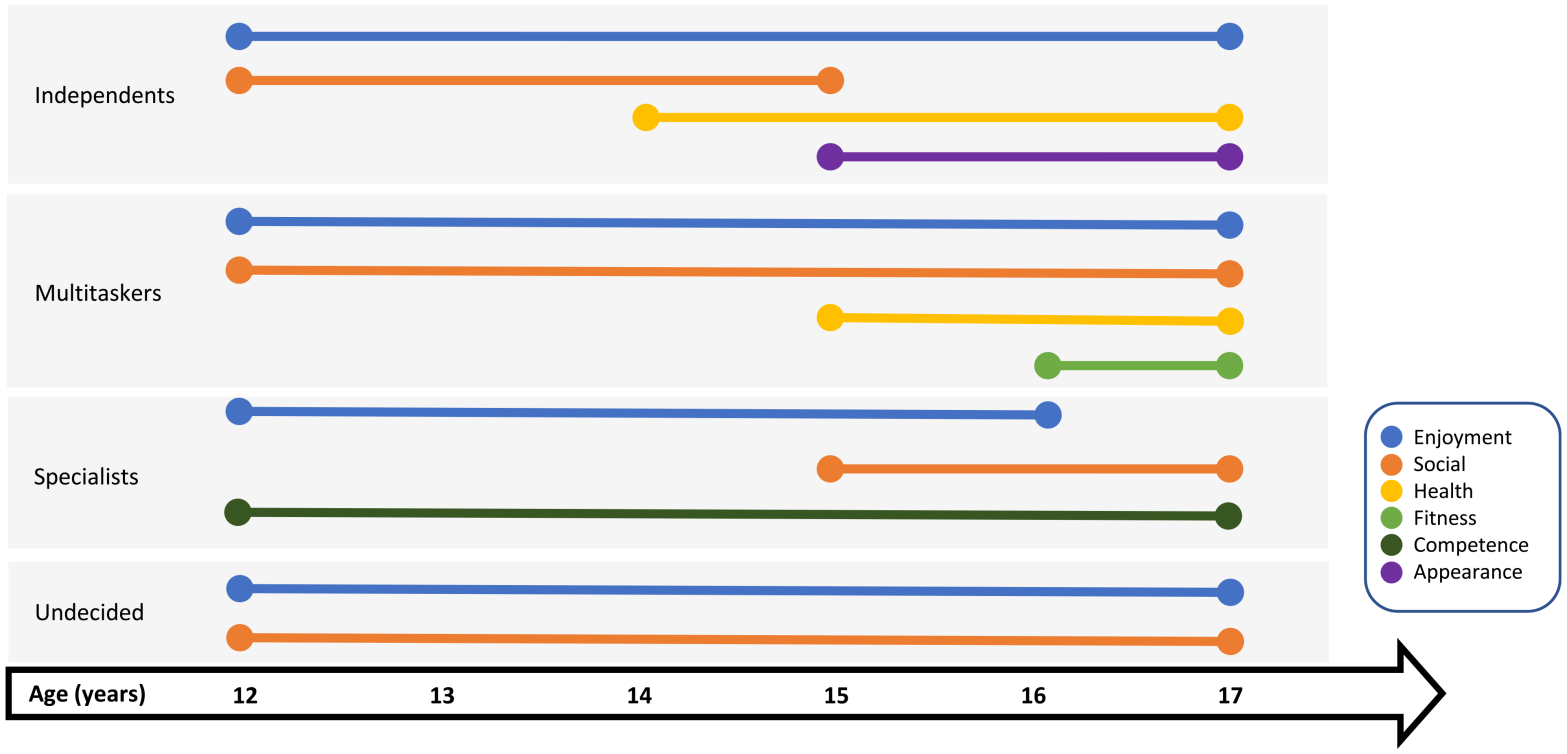
Predominant motives for participating in physical activity by age and across physical activity profiles.

**Figure 2:**
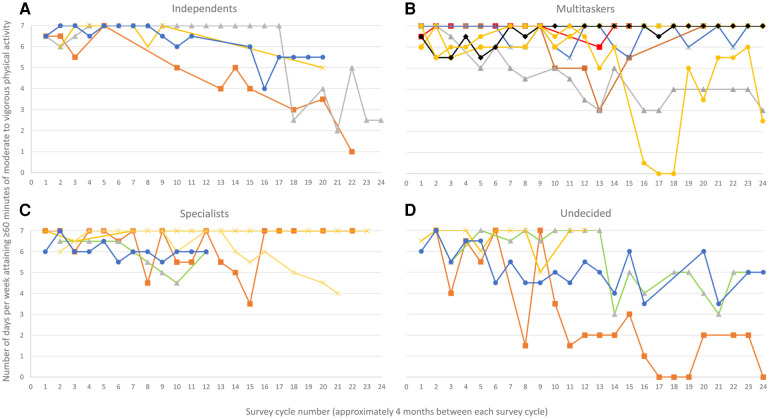
Self-reported physical activity level of participants across the 24 cycles of quantitative data collection during adolescence (each line represents a distinct participant. Losses to follow up occurred for the quantitative component of the study but all participants were retained for the qualitative component).

The analysis of participants' interview data led to the identification of four distinct PA profiles that captured participants’ unique PA trajectories and experiences through adolescence (Table 1). PA profiles, namely Independents, Multitaskers, Specialists, and Undecided, could be distinguished based on key characteristics and three themes, notably motives for PA, facilitators of participation, and barriers faced to remain physically active. Enjoyment, or “*because it's fun*”, was expressed as a primary motive for PA participation across all profiles in the first years of follow-up. As such, there is more focus on motives that further allowed distinguishing differences across profiles and on how motives for PA changed within each profile during adolescence (Figure 1). Similarly, the descriptions below center on distinctions in how participants from different profiles discussed facilitators and barriers, how they delt with them, and the influence they had on their PA participation. Whereas there were participants in all profiles who demonstrated maintenance or decline of PA, relatively more participants within the Specialists and Multitaskers profiles appeared to maintain high PA levels throughout adolescence (Figure 2).

**Table 1:** Key characteristics and sample quotes characterising each physical activity profile.

Independents *n = 4*	Multitaskers *n = 10*	Specialists *n = 5*	Undecided *n = 4*
– Seek autonomy. “*I like having my free time where I can do what I want.*” (Rachel) – Experience freedom through physical activity. “*I get the joy of feeling so free out in the water, like nothing is holding you back*” (David) – Physical activity is important, but not a predominant sphere of their lives. “*It might just be due to an interest in different things, but it was probably a couple weeks ago my buddy and I just went down to the park and just started playing tag for fun. So, I definitely still do enjoy, but it does seem to be a lot less present*.” (Isaac) – Affinity for the outdoors. “*It*'*s just nice to get outside, to not be trapped inside in front of a screen, just go out and enjoy the weather and stuff*.” (Isaac) – Preference for non-competitive physical activity. “*Having fun with friends is more important than winning or losing*.” (David)	– Love for multiple physical activities. “*I couldn*'*t (choose only one activity), because I really like being physically active and just doing one thing… well, I would get really bored to only do that thing*.” (Rob) – Being physically active is part of their identity. “*Physical activity is like my life. I couldn*'*t live without physical activity*” (Justine) – Value of social connections. “*If I didn*'*t have friends on the team, I probably wouldn't play*.” (Jason) – Support from parents. “*My parents are super active, both of them. Since I was young, they encouraged me to do sports and stay active*.” (Julia) – Aim for enjoyment. “*I wanted a year to really have fun*” (Rob)	– Highly invested in one sport. “*I feel that it*'*s important to have (…) one sport that you're really good at*.” (Sophie) – Would be lost without this physical activity. “*I just feel like I*'*ve been doing it for so long like I don't know what else I'd do if I didn't play*.” (Sophie) – Passion for one activity. “*I just love [Activity X]. It is kind of like your vitamin everyday it*'*s some people have to take vitamins to make them feel better well for me it's I have to Play [X] to make me feel better. So, I don't know how it works*.” (Kathy) – Aspire to be the best. “*I want to become better. I want to be the best in [Sport X].*” (Jeff) – Competitive attitude. “*I have goals and I know how to get there and what to do to get there and I*'*m doing it. If I keep going, then I have a good chance of making the team again*.” (Melanie)	– Physical activity is meant to give them something to do. “*Well, I don*'*t like to just stay inside my house and do nothing. So, it's pretty much to be able to do stuff instead of just staying inside*.” (Samantha) – Do not seek to be physically active (although they are). “*If the opportunity comes and people want to play, I*'*ll play*.” (Estelle) – Participate in physical activity on their own terms. “*I don*'*t want to do competitive [X], I just like the way that I feel when I do [X], it just makes me feel free to do what I want*” (Cody) – Losing interest. “*I think it*'*s just I got older, I got lazy kind of deal you know, when you're a teenager you just like* ‘*I don*'*t want to do anything.*’” (Christine)

### Physical activity profiles

#### Independents (*n* = 4)

*I can do whatever I want, just keep going until I want to stop and then go back*. (David)

The main defining characteristic of the four participants within the Independent profile is that as they got older they sought more autonomy from their PA pursuits. Feeling like they had a say in how and when they participated in physical activities was important to them. During their childhood and early adolescence, Independents mentioned often seizing opportunities to take part in a wide variety of physical activities. Through these exposures, they discuss developing a preference for non-competitive activities which eventually led them to seek out physical activities that could be performed individually and that are also typically unorganised.

*I like having my free time where I can do what I want and if I want to go to the gym, well, that's on my time, and I can decide when I go to the gym. Compare that to soccer - you have a fixed schedule and that's what it is*. (Rachel)

They reveal that they enjoyed not being fully committed to a given sport or PA as it allowed for the flexibility to try different physical activities when the opportunity presented itself.

*(…)there's more time to do other fun stuff… Social things, other sports too*. (David)

In general, even if they reported engaging in various types of activities, Independents displayed a particular affinity for outdoor physical activities as these provided them with the feelings of freedom and independence which they craved.

*We always try to go to cool places to go like swimming, go to the falls. Sometimes we hike to go swimming*. (David)

Activities during later adolescence also required minimal financial commitments, which proved an important determinant for sustaining participation over time. Beyond cost, time also became a challenge as participants in this profile started prioritizing school, friends, and work, which may be interpreted as a change in social roles as they aged. However, participants explained organizing their physical activities to maximize time spent with friends, while remaining physically active. This also facilitated maintenance of PA for them in later years.

In the later stages of data collection (ages 15–17), Independents progressively started to identify health outcomes as motives for being physically active. They reported recognizing, for example, that participation in PA contributed positively to their mental health. Engaging in PA with the aim of maintaining or improving their physical appearance also became a more prominent motive for some participants in this profile in later years.

*I'm going to Cuba next year and I kind of want to look good. So, I also do it for appearance reasons. Like, I'm not going to say: “Oh, I'm perfectly fine with my body.” No. I want to lose weight. Everyone has at least something they're self-conscious about, and that’s my thing. I gained weight and I really don't like it*. (Laura)

#### Multitaskers (*n* = 10)

*I play a lot of sports and I'm good at all sports*. (Rob)

Multitaskers take part in many different sports throughout adolescence. Participants within this profile also adopt PA as a lifestyle. Justine expressed this viewpoint succinctly: “*Physical activity is like my life. I couldn't live without physical activity*”. To these participants, engaging in many physical activities appears to simply be innate as Elsa herself states: “*I don't know, I just want to move and do something.”* This behaviour is such an integral part of their identity that these participants are at a loss to imagine what their life would be like without PA.

Multitaskers' primary motives for participating in PA are enjoyment and social connections. They describe being supported in their activities by family and friends, who are role models, participate with them, and provide them with encouragement.

*I often practice with my mom. because she really likes [Sport X] and she really played it a lot*. (Jason)

Although many Multitaskers report engagement in individual physical activities, they generally favour team sports, which they tend to maintain throughout adolescence.

Even if this group's schedule is typically filled with organized sport commitments, Multitaskers, in the early years, describe filling the remainder of their time with non-organised PA as exemplified by Ryan: “*…for me, winter and summer, there's nothing to stop me. I’m always busy*.” Time management issues nevertheless arise with age as they pursue other interests and commitments, including schoolwork and employment. This eventually forces them to make decisions and quit some physical activities. The participants talked about how these decisions are sometimes complicated as they find themselves torn between activities they prefer and activities in which, from their perspective, they excel. Despite having to make these difficult decisions, Multitaskers discussed refusing to commit to only one sport as they do not want to compromise their freedom to take part in multiple sports. Indeed, some Multitaskers described times when they joined a lower-level team to avoid devoting all their free time to the increased demands associated with participation at the highest levels of competition within a given sport.

*Well, I want to still keep the summer to myself and do other sport camps. So, it’s also something to consider if I'm going to play in the [elite] team*. (Julien)

For some participants in this group, participation in many different sports does not deter them from aspiring to attain high levels in most of them. Some Multitaskers talked about setting high standards for themselves and their teams, without taking the fun out of PA, as was expressed by Julia: “*…it's like in tournaments, in finals, you’re all stressed and you can't wait to play and it's super exciting*”.

For these participants, being with friends and feeling accepted within a team were important determinants of participation in the early years. This evolved later in adolescence to wanting to be on teams that shared the same values and a similar passion for sport. As Justine explained: “*It's really important cause if there's tension on the court it's not going to go well.”* Over the years, Multitaskers also talked about noticing the positive effects that PA had on their fitness and general health, and this became an additional reason for them to maintain an active lifestyle.

#### Specialists (*n* = 5)

*I want to be the best I can at one thing. I want to always improve and always become better and play better every day. That’s what inspires me*. (Jeff)

Specialists aspire to be the best. The sport in which they strive to be the best does not appear to matter excessively, as long as it allows them to pursue a path of high-level performance. Participants in this group mention being drawn during childhood to specialise and be entirely invested in attaining the top level of their sport.

*I feel that it's important to have skills… like one sport that you're really good at. Cause lots of people play different sports but they always have one sport that is their main sport, and it's really cool to know that you're really really good at a sport*. (Sophie)

While Specialists might still participate in other physical activities, they describe generally doing so to contribute to the development of competencies for their main sport. Since their main sport requires an important time-commitment and occupies a large portion of their life, it often becomes part of their identity. Their sport is who they are (i.e., a skater, a dancer, etc.). It is therefore difficult for members of this profile to face unexpected and prolonged interruptions when it comes to participating in their main sport.

*I'm not used to not playing it. It's kind of weird, I'm not going to a tournament this weekend, I don't have to rush to practice this weekend. I don't know what to do with my life*. (Melanie)

Specialists display the personality traits of high achievers. Coincidentally, some participants in this group aspire to reach the highest level of performance in their activity. As their primary motive for PA lies within the development of competencies, Specialists are typically ready to do whatever it takes to improve their ability to perform. For example, when asked what he would be willing to do in order to reach a professional level in his sport, Jeff replied:

*Everything. Anything. I will do anything I need to do. If I need to go hit balls everyday, play everyday… Even if it rains, if it's cold, I'll go. It's pretty much everything. It would be… it would be my all-time goal to get there*. (Jeff)

The pursuit of their goals is facilitated by access to a very strong support network and what seems like unlimited resources located within their immediate environment. Most specifically, these participants explain how their parents eliminated financial barriers and are role models, cheerleaders and confidants.

Concurrently, as they got older, Specialists also reported many barriers, including losing interest and struggling to find a balance with their growing responsibilities and other interests. Participants in this group also endured frequent psychological stresses as they faced pressure to succeed, both from within themselves and from their entourage. Their involvement in high-performance sports also increased the likelihood that they would be exposed to interference during team selection, negative experiences with coaches or challenging interactions with other athletes. This led some Specialists to eventually convey becoming disenchanted with their main activity. For some participants, negative experiences or peripheral aspects related to their sport participation led to discontentment. For example, Melanie explains how she felt after being invited to play for a team, only to be asked to leave several days later because it appears too many players were already on the team's roster:

*I was mad and my whole team was mad. Everyone was just mad. He [the coach] is not winning any friends, I don’t think. Not very many people will play for him again*. (Melanie)

However, Specialists who developed discontentment reported recognizing that it was due to external factors, and they continued to have appreciation for their activity. Some participants in this group therefore transition to another PA for which they believe their skills will be transferable (as they maintain high-performance aspirations), but in which negative aspects are expected to be less prevalent. However, not all participants in this profile experienced disenchantment or discontentment. For Jeff, the process of specializing into a sport came gradually; after initially playing multiple sports concurrently, he progressively dropped activities that interfered with the time commitments required for his main sport (“… *I want to become better. I want to be the best in [Sport X]. I want to stop [Sport Y] and start doing more for [Sport X].*”). This approach might have contributed to a more successful experience as this participant showed no signs of burn-out or lack of passion for the main sport after many years of participation.

#### Undecided (*n* = 4)

*It just fills my time and just gives me something to do*. (Christine)

For their part, participants classified in the Undecided profile discuss engaging in PA to occupy their free time. They recognize that other people often draw them into PA, and they enjoy taking part when with parents or friends.

*If it weren't for my parents being active like they are, I think I would never have done the things I do today*. (Samantha)

Participants in this profile do not seek to adhere to societal or group norms and therefore keep an open mind when presented with chances to try different things: “*I don't know why they run from the other side and they do their flips and stuff. But I usually just jump in a few times and then I just tend to stay in the water and swim.*” (Christine). The Undecided favour physical activities that are non-team-based, non-competitive and unorganised. When taking part in PA, they do so on their own terms and at their own pace. Nevertheless, they tend not to seek or create their own opportunities to engage in PA, but instead describe partaking in activities that are presented to them, if and when they see fit.

*I'll go on a bike ride if everyone is going on a bike ride and it's an activity that we do or, I will go on a walk if everyone is going for a walk. Like, I'll play catch if everyone is playing. If the opportunity comes and people want to play, I'll play*. (Estelle)

When the Undecided take part in an activity, they generally do so without performance objectives in mind. Coincidentally, they present a low level of commitment to their physical activities and any barrier, however small, threatens to disrupt their participation at any time. Since PA, from their perspective, is intended to give them something to do, their participation is centered on leisure and is typically characterized by a relatively low level of intensity.

*When it comes to sports, I say “It*’*s just sports, what*’*s the big deal? You lost, who cares?”. It's just a sport…* (Cody)

In contrast with the other three profiles, the Undecided identified a relatively larger number of barriers to PA. Most particularly, they often did not receive emotional or tangible support for PA from their parents.

*I didn't want to go by myself because it was scary. My mom definitely couldn't go because she was injured, and my dad was away and no one else skates*. (Christine)

Several participants shared experiences that left them with a dislike for sports, mostly expressed in relation to team sports. Some were hesitant to participate again in this type of activity for fear of experiencing exclusion, intimidation or bullying from teammates, as they had in the past.

*I'm still not sure with the teams that are really organized because sometimes the students who participate just want to win, and they don't really care about making connections or being nice to others*. (Estelle)

Participants in this profile also expressed a sense of loathing for the increasing level of commitment associated with sports with age.

*What’s fun is if I'm not interested in doing it for say one or two weeks because I'm just too tired or I have too many things to do and I don't feel like it, like I don't need to. That’s something that was really annoying with organized sports and that’s one of the reasons why I'm a little hesitant to go somewhere organized*. (Estelle)

Despite their lack of intrinsic interest for sports, the Undecided remain open to other forms of PA. Toward the end of follow-up, those who engaged most in PA endorsed outdoor-based activities that offered the flexibility to be practiced alone or with others. This is consistent across participants in this profile and across time as they describe their most positive PA experiences in the context of outdoors and unorganised activities.

*I feel like, I hear bird sounds and everything like the nature around me, and it’s not just being on electronics. It takes me out of those things*. (Samantha)

## Discussion

This is the first study aiming to qualitatively identify and describe different groups of initially physically active adolescents over six years. With its sequentially repeated interviews, the current study advances our understanding by providing unique insight into the underlying reasons for which youth report participating in different leisure-based physical activities and offer an insider's view of the emotions and feelings experienced through this participation. Our results highlight that initially physically active individuals can present very diverging experiences with PA during adolescence. These differences in experiences, as well as in motives, facilitators and barriers allowed to distinguish adolescents across four emerging PA profiles, which we identified as Independents, Multitaskers, Specialists, and Undecided.

Following identification of these PA profiles, it was noted that some of them share important points of similarity with the pathways of sport participation presented in the Developmental model of sport participation (DMSP) (56). For example, in the early years of follow-up, Independents and Multitaskers were consistently involved in multiple activities, akin to the sampling and recreational years described in the DMSP. Consistent with suggestions in the DMSP, this multi-sport exposure was associated with opportunities for participants to identify which types of activities they preferred and then sustain such activities going forward. Also, in agreement with the DMSP, later specialization in a given activity was associated with more positive experiences. However, our findings diverged from the DMSP in one area. While Independents and Undecided were involved in different sports during childhood and early adolescence, their PA eventually became exclusively non-sport based over time, a transition the DMSP would characterise as “dropouts”. Yet, labelling all these participants as dropouts would be inaccurate for youth who remained physically active (57). Representing non-sport based PA appears relevant even for sport participation models since this behaviour often takes root in early experiences with sports.

The finding that enjoyment is an important determinant of PA participation across all profiles and throughout adolescence is consistent with many quantitative studies (58–60) and corroborates suggestions that PA interventions should be focused on enjoyment (61, 62). However, developing enjoyable PA experiences may be challenging since our results highlight that enjoyment takes different meanings across different profiles. For Independents, enjoyment relates to experiencing freedom; for Multitaskers, it is about experiencing movement and camaraderie; for Specialists, it means achieving successes; and for the Undecided, it equates to offering a positive distraction. Our results indicate that tailored approaches to the promotion of PA may be the most promising method to effectively retain PA participation over time, although likely more costly and resource intensive.

Profile-specific interventions for promoting PA are also important given that other motives for taking part in PA were among the key characteristics allowing to distinguish across the different PA profiles. For example, participating in sports and PA to spend time with loved ones among the Undecided and to develop and maintain social affiliations among Multitaskers were important motives throughout the study for these profiles. In contrast, the social aspect of PA was only an important motive in the early years among Independents and gained in importance among Specialists in later years. Furthermore, none of the profiles engaged in PA for fitness or health motives at the beginning of adolescence, but these became goals underlying participation in later years among Independents and Multitaskers. Finally, being active in order to improve skills and to seek the excitement of competitions were participation objectives among Specialists, which distinguishes them from the Undecided and Independents, for whom competence-related aspects of sports were deterrents to participation. Taking into account the varied reasons why youth choose to take part in sport and PA appears fundamental to the success of initiatives that promote PA. This is consistent with the recommendation from Pedersen et al. (63) to use group-specific knowledge regarding motives for participation in PA in order to inform health promotion initiatives.

The social aspect of PA was discussed as a motive for participation, but the latter also acted as a predominant facilitator in maintaining participation, especially when participants experienced social connections that they found to be enjoyable. However, as discussed in previous studies (64), social connections can also have an adverse effect on long term participation. In cases where participants experienced negative talk, discrimination or bullying from teammates, as well as favoritism or abusive behaviours from coaches, participants from all profiles either discontinued their participation or considered dropping out of PA. Our data align with previous suggestions that social environments that are favourable to sustaining youth participation in PA are those that provide participants with a sense of closeness with others, a feeling of sharing a similar commitment and one of mutual complementarity across members of a group (65, 66). In this sense, our results are consistent with SDT in suggesting that ongoing efforts to promote relatedness through positive, inclusive, and ethical sport and PA experiences for all must therefore be sustained. However, not as much support emerged from our results for the importance to promote the other SDT basic psychological needs of autonomy and competence. This should nevertheless not be interpreted as incompatibility with the SDT since the inductive analytical approach did not intend to test a theory.

Participants who experienced co-participation in PA with a parent reported its positive impact on their PA participation trajectory. Consistent with recent findings, parental support also had positive effects when taking the form of providing transportation to and being present during physical activities (67). There were important contrasts in level of parental support reported by participants among the different profiles. Most importantly, most Multitaskers and Specialists gave the impression of benefiting from limitless support from their parents, whether it be through encouragements, co-participation, transportation, watching, or mentoring, whereas a majority of the Undecided appeared not to have access to such parental engagement. These differences likely influenced participants' PA trajectory and point to the importance of parental support for their children's continued participation in PA.

## Strengths and limitations of the study

Strengths of this study include the six years of follow-up with yearly interviews. Also, the use of an inductive and collaborative approach for the identification of PA profiles allowed them to be data-driven. The later use of deductive analysis facilitated linking the data with theoretical models. Further, this study has the advantage of not being tied to a specific sport or PA, which allows for comparisons to be drawn with general sport participation models. The analytical procedure also included multiple checks among analysts and peers. Direct quotes were used to authentically represent the data. The study's rigor was also supported by the establishment of a rapport between interviewers and participants both during the coordination of the study and the in -person interviews. Regarding data saturation, a review of the analysis reveals that omitting the last two years of interview data does not lead to any noteworthy changes to the themes or to the four PA profiles identified herein. This lends confidence that data appropriately captured the breadth of experiences that initially physically active individuals might have throughout adolescence. Limitations nevertheless include that the large number of interviews makes it impossible to explore all facets of the data in one manuscript. Furthermore, given the aim of the study, only experiences of adolescents who were initially physically active, through recreational physical activities, are represented. Generalizability of results is also limited by the small sample size from a single geographic location. It must also be recognized that typologies can vary across social and cultural contexts, meaning that external validation is warranted to confirm the profiles proposed and described herein. Finally, the self-reported questionnaire used to document the PA level of participants is subject to social desirability.

## Conclusion

The profiles identified provide an understanding of important differences across the PA experiences of different groups of youth. Results highlight that different groups of adolescents take part in PA for very different reasons and that they face barriers to participation differently. Participants in the different profiles also benefit from different facilitators, which influences their experience and participation trajectory. These results underscore the importance of approaching the promotion of PA with approaches specifically tailored to different sub-groups of adolescents.

## Data Availability

The raw data supporting the conclusions of this article will be made available by the authors, without undue reservation.

## References

[1] Guthold R Stevens GA Riley LM Bull FC. Global trends in insufficient physical activity among adolescents: a pooled analysis of 298 population-based surveys with 1·6 million participants. Lancet Child Adolesc Health. (2020) 4:23–35. 10.1016/S2352-4642(19)30323-2 31761562 PMC6919336

[2] Mäkelä S Aaltonen S Korhonen T Rose RJ Kaprio J. Diversity of leisure-time sport activities in adolescence as a predictor of leisure-time physical activity in adulthood. Scand J Med Sci Sports. (2017) 27:1902–12. 10.1111/sms.12837 28106293 PMC5806530

[3] Bélanger M Sabiston CM Barnett TA O’Loughlin E Ward S Contreras G Number of years of participation in some, but not all, types of physical activity during adolescence predicts level of physical activity in adulthood: results from a 13-year study. Int J Behav Nutr Phys Act. (2015) 12(76). 10.1186/s12966-015-0237-x 26058349 PMC4464637

[4] Batista MB Romanzini CLP Barbosa CCL Blasquez Shigaki G Romanzini M Ronque ERV. Participation in sports in childhood and adolescence and physical activity in adulthood: a systematic review. J Sports Sci. (2019) 37:2253–62. 10.1080/02640414.2019.1627696 31179841

[5] Logan K Lloyd RS Schafer-Kalkhoff T Khoury JC Ehrlich S Dolan LM Youth sports participation and health status in early adulthood: a 12-year follow-up. Prev Med Rep. (2020) 19(101107). 10.1016/j.pmedr.2020.101107 32477851 PMC7248647

[6] Kwon S Letuchy EM Levy SM Janz KF. Youth sports participation is more important among females than males for predicting physical activity in early adulthood: Iowa bone development study. Int J Environ Res Public Health. (2021) 18:1328. 10.3390/ijerph18031328 33540518 PMC7908602

[7] Doré I O’Loughlin JL Schnitzer ME Datta GD Fournier L. The longitudinal association between the context of physical activity and mental health in early adulthood. Ment Health Phys Act. (2018) 14:121–30. 10.1016/j.mhpa.2018.04.001

[8] Guddal MH Stensland SØ Småstuen MC Johnsen MB Zwart J-A Storheim K. Physical activity and sport participation among adolescents: associations with mental health in different age groups. Results from the young-HUNT study: a cross-sectional survey. BMJ Open. (2019) 9(e028555). 10.1136/bmjopen-2018-028555 31488476 PMC6731817

[9] Rangul V Bauman A Holmen TL Midthjell K. Is physical activity maintenance from adolescence to young adulthood associated with reduced CVD risk factors, improved mental health and satisfaction with life: the HUNT study Norway. Int J Behav Nutr Phys Act. (2012) 9(144). 10.1186/1479-5868-9-144 23241306 PMC3541207

[10] Silva DR Werneck AO Collings PJ Fernandes RA Barbosa DS Ronque ER V Physical activity maintenance and metabolic risk in adolescents. J Public Health (Bangkok). (2018) 40:493–500. 10.1093/pubmed/fdx077 28927241

[11] Torres W Cayres-Santos SU Urban JB de Moraes-Chagas LG Christofaro DGD Turi-Lynch BC Participation in non-professional sports and cardiovascular outcomes among adolescents: ABCD growth study. Matern Child Health J. (2020) 24:787–95. 10.1007/s10995-020-02919-1 32323117

[12] Niehoff NM White AJ Sandler DP. Childhood and teenage physical activity and breast cancer risk. Breast Cancer Res Treat. (2017) 164:697–705. 10.1007/s10549-017-4276-7 28500399 PMC5553118

[13] Hidayat K Zhou H-J Shi B-M. Influence of physical activity at a young age and lifetime physical activity on the risks of 3 obesity-related cancers: systematic review and meta-analysis of observational studies. Nutr Rev. (2020) 78:1–18. 10.1093/nutrit/nuz024 31393566

[14] Gunter KB Almstedt HC Janz KF. Physical activity in childhood may be the key to optimizing lifespan skeletal health. Exerc Sport Sci Rev. (2012) 40:13–21. 10.1097/JES.0b013e318236e5ee 21918458 PMC3245809

[15] Martins J Marques A Sarmento H da Costa F C. Adolescents’ perspectives on the barriers and facilitators of physical activity: a systematic review of qualitative studies. Health Educ Res. (2015) 30:742–55. 10.1093/her/cyv042 26324394

[16] Martins J Costa J Sarmento H Marques A Farias C Onofre M Adolescents’ perspectives on the barriers and facilitators of physical activity: an updated systematic review of qualitative studies. Int J Environ Res Public Health. (2021) 18:4954. 10.3390/ijerph18094954 34066596 PMC8125166

[17] Drew K Morris R Tod D Eubank M. A meta-study of qualitative research on the junior-to-senior transition in sport. Psychol Sport Exerc. (2019) 45(101556). 10.1016/j.psychsport.2019.10155634421367

[18] Franck A Stambulova NB. Individual pathways through the junior-to-senior transition: narratives of two Swedish team sport athletes. J Appl Sport Psychol. (2020) 32:168–85. 10.1080/10413200.2018.1525625

[19] Ryba TV Ronkainen NJ Douglas K Aunola K. Implications of the identity position for dual career construction: gendering the pathways to (dis)continuation. Psychol Sport Exerc. (2021) 53(101844). 10.1016/j.psychsport.2020.10184433110396

[20] Kristiansen E MacIntosh EW Parent MM Houlihan B. The youth Olympic games: a facilitator or barrier of the high-performance sport development pathway? Eur Sport Manag Q. (2018) 18:73–92. 10.1080/16184742.2017.1383499

[21] Ryba TV Stambulova NB Selänne H Aunola K Nurmi J-E. “Sport has always been first for me” but “all my free time is spent doing homework”: dual career styles in late adolescence. Psychol Sport Exerc. (2017) 33:131–40. 10.1016/j.psychsport.2017.08.011

[22] Casey MM Eime RM Payne WR Harvey JT. Using a socioecological approach to examine participation in sport and physical activity among rural adolescent girls. Qual Health Res. (2009) 19:881–93. 10.1177/1049732309338198 19556398

[23] Gardner SM Komesaroff P Fensham R. Dancing beyond exercise: young people's experiences in dance classes. J Youth Stud. (2008) 11:701–9. 10.1080/13676260802393294

[24] Wylleman P Lavallee D. A developmental perspective on transitions faced by athletes. In: Weiss MR, editor. Developmental Sport and Exercise Psychology: A Lifespan Perspective. Morgantown, WV: Fitness Information Technology (2004). p. 507–27.

[25] Bélanger M Casey M Cormier M Laflamme Filion A Martin G Aubut S Maintenance and decline of physical activity during adolescence: insights from a qualitative study. Int J Behav Nutr Phys Act. (2011) 8(117). 10.1186/1479-5868-8-117 22017754 PMC3215642

[26] Ryan RM Deci EL. Self-determination theory and the facilitation of intrinsic motivation, social development, and well-being. Am Psychol. (2000) 55:68–78. 10.1037/0003-066X.55.1.68 11392867

[27] Fortier MS Vallerand RJ Briere NM Provencher PJ. Competitive and recreational sport structures and gender: a test of their relationship with sport motivation. Int J Sports Psychol. (1995) 26:24–39.

[28] McAuley E Tammen VV. The effects of subjective and objective competitive outcomes on intrinsic motivation. J Sport Exerc Psychol. (1989) 11:84–93. 10.1123/jsep.11.1.84

[29] Martin JJ Gill DL. The relationships of competitive orientations and self-efficacy to goal importance, thoughts, and performance in high school distance runners. J Appl Sport Psychol. (1995) 7:50–62. 10.1080/10413209508406300

[30] Graham TR Kowalski KC Crocker PRE. The contributions of goal characteristics and causal attributions to emotional experience in youth sport participants. Psychol Sport Exerc. (2002) 3:273–91. 10.1016/S1469-0292(01)00006-1

[31] Ullrich-French S Smith AL. Perceptions of relationships with parents and peers in youth sport: independent and combined prediction of motivational outcomes. Psychol Sport Exerc. (2006) 7:193–214. 10.1016/j.psychsport.2005.08.006

[32] Fraser-Thomas J Côté J Deakin J. Understanding dropout and prolonged engagement in adolescent competitive sport. Psychol Sport Exerc. (2008) 9:645–62. 10.1016/j.psychsport.2007.08.003

[33] Leff SS Hoyle RH. Young athletes’ perceptions of parental support and pressure. J Youth Adolesc. (1995) 24:187–203. 10.1007/BF01537149

[34] Butcher JL Koenraad J Johns DP. Withdrawal from competitive youth sport: a retrospective ten-year study. J Sport Behav. (2002) 25:145–63.

[35] Vlachopoulos SP Neikou E. A prospective study of the relationships of autonomy, competence, and relatedness with exercise attendance, adherence, and dropout. J Sports Med Phys Fitness. (2007) 47:475–82. 18091690

[36] Jõesaar H Hein V. Psychosocial determinants of young athletes’ continued participation over time. Percept Mot Skills. (2011) 113:51–66. 10.2466/05.06.13.PMS.113.4.51-66 21987909

[37] Standage M Gillison FB Ntoumanis N Treasure DC. Predicting students’ physical activity and health-related well-being: a prospective cross-domain investigation of motivation across school physical education and exercise settings. J Sport Exerc Psychol. (2012) 34:37–60. 10.1123/jsep.34.1.37 22356882

[38] Withall J Jago R Fox KR. Why some do but most don’t. Barriers and enablers to engaging low-income groups in physical activity programmes: a mixed methods study. BMC Public Health. (2011) 11(507). 10.1186/1471-2458-11-507 21711514 PMC3141466

[39] Cresswell SL Eklund RC. Athlete burnout: a longitudinal qualitative study. Sport Psychol. (2007) 21:1–20. 10.1123/tsp.21.1.1

[40] Torregrosa M Ramis Y Pallarés S Azócar F Selva C. Olympic athletes back to retirement: a qualitative longitudinal study. Psychol Sport Exerc. (2015) 21:50–6. 10.1016/j.psychsport.2015.03.003

[41] Podlog L Eklund RC. A longitudinal investigation of competitive athletes’ return to sport following serious injury. J Appl Sport Psychol. (2006) 18:44–68. 10.1080/10413200500471319

[42] Beck CT, editor. Routledge International Handbook of Qualitative Nursing Research. London: Routledge (2013). 10.4324/9780203409527

[43] Doyle L McCabe C Keogh B Brady A McCann M. An overview of the qualitative descriptive design within nursing research. J Res Nurs. (2020) 25:443–55. 10.1177/1744987119880234 34394658 PMC7932381

[44] Kim H Sefcik JS Bradway C. Characteristics of qualitative descriptive studies: a systematic review. Res Nurs Health. (2017) 40:23–42. 10.1002/nur.21768 27686751 PMC5225027

[45] Sandelowski M. Whatever happened to qualitative description? Res Nurs Health. (2000) 23:334–40. 10.1002/1098-240X(200008)23:4<334::AID-NUR9>3.0.CO;2-G 10940958

[46] Sandelowski M. What’s in a name? Qualitative description revisited. Res Nurs Health. (2010) 33:77–84. 10.1002/nur.20362 20014004

[47] Prochaska JJ Sallis JF Long B. A physical activity screening measure for use with adolescents in primary care. Arch Pediatr Adolesc Med. (2001) 155(5):554–9. 10.1001/archpedi.155.5.554 11343497

[48] Caspersen CJ Powell KE Christenson GM. Physical activity exercise and physical fitness: definitions and distinctions for health-related research synopsis. Public Health Rep. (1985) 100:126–31. 3920711 PMC1424733

[49] Khan KM Thompson AM Blair SN Sallis JF Powell KE Bull FC Sport and exercise as contributors to the health of nations. Lancet. (2012) 380:59–64. 10.1016/S0140-6736(12)60865-4 22770457

[50] Miles MB Huberman AM. Qualitative data analysis: an expanded sourcebook. J Environ Psychol. (1994) 14:336–7. 10.1016/S0272-4944(05)80231-2

[51] Sabiston CM McDonough MH Sedgwick WA Crocker PRE. Muscle gains and emotional strains: conflicting experiences of change among overweight women participating in an exercise intervention program. Qual Health Res. (2009) 19:466–80. 10.1177/1049732309332782 19299753

[52] Creswell JW Poth CN. Qualitative Inquiry & Research Design: Choosing Among Five Approaches. 4th ed. Los Angeles: SAGE Publications, Inc. (2018). 10.46743/2160-3715/2019.4294

[53] Weston C Gandell T Beauchamp J McAlpine L Wiseman C Beauchamp C. Analyzing interview data: the development and evolution of a coding system. Qual Sociol. (2001) 24:381–400. 10.1023/A:1010690908200

[54] Grossoehme D Lipstein E. Analyzing longitudinal qualitative data: the application of trajectory and recurrent cross-sectional approaches. BMC Res Notes. (2016) 9(136). 10.1186/s13104-016-1954-1 26936266 PMC4776420

[55] McDonough MH Sabiston CM Ullrich-French S. The development of social relationships, social support, and posttraumatic growth in a dragon boating team for breast cancer survivors. J Sport Exerc Psychol. (2011) 33:627–48. 10.1123/jsep.33.5.627 21984639

[56] Côté J Baker J Abernethy B. Practice and play in the development of sports expertise. Handb Sports Psychol. (2007) 3:184–202. 10.1002/9781118270011.ch8

[57] Gallant F Bélanger M. Empirical support for the tenets of sport participation and physical activity-based models: a scoping review. Front Sports Act Living. (2021) 3. 10.3389/fspor.2021.741495 34723180 PMC8552970

[58] Nader PA Gaudet J Brunet J Gunnell KE Doré I Sabiston CM Associations between physical activity motives and trends in moderate-to-vigorous physical activity among adolescents over five years. J Sports Sci. (2021) 39:2147–60. 10.1080/02640414.2021.1923203 34259129

[59] Dishman RK Saunders RP McIver KL Dowda M Pate RR. Construct validity of selected measures of physical activity beliefs and motives in fifth and sixth grade boys and girls. J Pediatr Psychol. (2013) 38:563–76. 10.1093/jpepsy/jst013 23459310 PMC3716273

[60] Seghers J Vissers N Rutten C Decroos S Boen F. Intrinsic goals for leisure-time physical activity predict children’s daily step counts through autonomous motivation. Psychol Sport Exerc. (2014) 15:247–54. 10.1016/j.psychsport.2014.01.003

[61] van Sluijs EMF Kriemler S. Reflections on physical activity intervention research in young people—dos, don’ts, and critical thoughts. Int J Behav Nutr Phys Act. (2016) 13(25). 10.1186/s12966-016-0348-z 26892920 PMC4757975

[62] Lewis BA Williams DM Frayeh A Marcus BH. Self-efficacy versus perceived enjoyment as predictors of physical activity behaviour. Psychol Health. (2016) 31:456–69. 10.1080/08870446.2015.1111372 26541890 PMC4769927

[63] Pedersen MRL Hansen AF Elmose-Østerlund K. Motives and barriers related to physical activity and sport across social backgrounds: implications for health promotion. Int J Environ Res Public Health (2021) 18:5810. 10.3390/ijerph18115810 34071630 PMC8198157

[64] Sheridan D Coffee P Lavallee D. A systematic review of social support in youth sport. Int Rev Sport Exerc Psychol. (2014) 7:198–228. 10.1080/1750984X.2014.931999

[65] Jowett S Timson-Katchis M. Social networks in sport: parental influence on the coach-athlete relationship. Sport Psychol. (2005) 19:267–87. 10.1123/tsp.19.3.267

[66] Jowett S Shanmugam V Caccoulis S. Collective efficacy as a mediator of the association between interpersonal relationships and athlete satisfaction in team sports. Int J Sport Exerc Psychol. (2012) 10:66–78. 10.1080/1612197X.2012.645127

[67] Doggui R Gallant F Bélanger M. Parental control and support for physical activity predict adolescents’ moderate to vigorous physical activity over five years. Int J Behav Nutr Phys Act. (2021) 18(43). 10.1186/s12966-021-01107-w 33752697 PMC7986262

