# Supplementary material for: Identification of distinct physical activity profiles through adolescence: a longitudinal qualitative description study
Source: Front Sports Act Living. 2024 Aug 22;6:1230999. doi: 10.3389/fspor.2024.1230999 (PMC11374625; doi:10.3389/fspor.2024.1230999)
Supplement: Supplementary file 1 [file Datasheet1.docx]

**Interview guide**

**BASELINE INTERVIEW**

Goal: To describe experience of physical activity as lived through it (what, when, where, with whom, how, including feelings, mood, emotions, sounds, smells, how body feels) by collecting stories, anecdotes, experiences, incidents, adventures, happenings etc. participant has had in the past year with a sport/physical activity.

ICE BREAKING QUESTIONS. Ex. What is your favorite thing to do when you’re not in school?

1. Please tell me the different physical activities you do [use photos to help remember]. How often do you do it? How long have you been doing it? How did you get started? How did you realize that it was the right activity for you?
2. Could you describe for me the last times you did X? One of the best times you had doing X? Worst times? [What happened? With whom? When? Where? What made that time so good/so bad?]
3. For you, what is the best thing about X? What is the worst thing?
4. How do you feel when you do X? What would happen if you couldn’t do X anymore? What do you get out of doing X? How important is it for you to do X? If you wanted to quit, what would happen? Could you do it?
5. What is it like to [be part of a team? play/do activities outdoors/on your own? take part in classes/competitions?]
6. On a scale of 1 to 10, how good would you say you are you at X? What would you say are your strengths/things you are good at in X? Anything you would like to get better at? How would you go about it? Who helps you develop your skills? Who encourages you? What do you think other people would say about you [friends, teammates, family]?
7. The activities that you do… The outings, classes, competitions… Who decides - when, which ones, what events?
8. What physical activities do you see yourself doing this summer? Next year? Any activities that you plan to try or would like to try? How would go about it?
9. Is there anything you would like to share with us?

Probes and prompts

Can you tell me a bit more about that?

What do you mean by…?

What makes you say that?

How did you feel about that?

What is it like to…?

Can you give me an example?

So you say that…? [repeat a last sentence in a questioning tone]

**Interview guide**

**FOLLOW-UP INTERVIEW**

This is a semi-structured interview. There are key questions to address (boxed and bolded). Whenever necessary, you should ask additional questions to further your understanding of the experience of physical activity participation.

Goals: to describe experiences of physical activity as lived by a youth (what, when, where, with whom, how, including feelings, mood, emotions, sounds, smells, how the body feels) by collecting stories, anecdotes, experiences, incidents, adventures, happenings etc. that they have had in the past year with a sport/physical activity.

To understand the evolution of their physical activity participation.

Probes and prompts to help participants elaborate

Can you tell me a bit more about that?

What do you mean by…?

What makes you say that?

How did you feel about that?

What is it like to…?

Can you give me an example?

So you say that…? [Repeat a last sentence in a questioning tone]

**1. It is your (#) year at this school. How did it go? How was the transition (If new school)? How did this impact your PA participation?** [See where this goes first, then continue with next questions]

**2. Tell me about the different physical activities you did in the past year? Perhaps we can start with last summer and go through the seasons until now** [write down the different activities for each season]

**D. Is there anything that could have made you continue this activity (or these activities)?**

[If they say no]: **What makes you say that?**

[If they say yes]: **Like what?**

**7. What does doing X bring you? What makes you want to continue to do X? What do you think your participation in Activity X will look like after high school?**

**8. How do you think the importance you attribute to Activity X may change after high school?**

**Which ones take up most of your time?**

**3. Of these activities** [Read them]**, which ones are most important to you? How are decisions made about activities to prioritize? What makes you say that?**

**4. Which ones take up more of your time? If you could just do X, would you quit your other activities? What makes you say that?**

**5. Tell me about how you interact with others when you do X?**

**C. To help me understand what it was like to do this activity (those activities), was there a moment in the past year that made you think: “Ugh, I don’t want to do this anymore!” that you can share?**

**B. How did you go about making the choice to stop? Can you tell me more about the process?**

**B. To help me understand what it is like to do this activity (those activities), was there a moment in the past year that made you think: “Wow, this is why I do this?” that you can share?**

**C. On the contrary, was there a moment in the past year that made you think: “Ugh, why do I do this?” that you can share?**

**B. To help me understand what it is like to do this activity (those activities), do you have any stories you could share?**

**A. What brings you to do this activity (those activities)? What is it about it this activity (those activities) that makes you want to do it?**

***IF THEY’VE STARTED A NEW ACTIVITY (OR ACTIVITIES)***

i. How would you convince a friend to try it? What’s the best part?

ii. With whom do you do X?

iii. How did you get started? Who was involved in your decision to start X?

iv. Are there skills you want to develop or maintain by doing X? Which ones?

i. Can you tell me about one of the best times you had doing X? Worst time?

ii. What happened?

iii. What made that time so good/so bad?

iv. With whom? v. When? Where?

i. Anything you would like to get better at/work on?

ii. How would you go about it?

iii. What or who could help?

iv. What or whom encourages you to want to get better?

i. Are there any barriers you can identify? (Injuries, $, etc.)

ii. How do you think you will deal with these challenges?

***IF THEY’VE QUIT THEIR ACTIVITY (OR ACTIVITIES)***

i. Did something change in your life that made you want to quit?

ii. Did something change?

iii. What was the worst part?

i. Did you have friends that did it?

ii. Whose decision was it to quit?

iii. Did you talk to anyone about it before doing it?

i. What happened?

ii. What made that moment so bad?

iii. With whom?

iv. When? Where?

i. What were the parts of it that you really enjoyed?

***IF THEY’VE MAINTAINED THEIR ACTIVITY (OR ACTIVITIES)***

i. Did you increase or decrease participation? What makes you say that?

ii. What’s the best part? What makes you want to continue?

iii. Any changes in with whom you participate?

iv. Any changes in how you feel when participating? Which ones?

v. Are there any new skills you want to develop or maintain? Which ones?

i. What happened? ii. With whom? iii. When? Where?

i. Anything you would like to get better at/work on?

ii. How would you go about it?

iii. What or who could help?

iv. What or whom encourages you to want to get better?

i. Are there any barriers you can identify? (Injuries, $, new School, etc.)

ii. How do you think you will deal with these challenges?

**6. Last year you talked about X.** [Confirm what is observed by the answers in questions 2 to 5]

**Which ones take up most of your time?**

**A. What made you quit this activity (those activities)? How did it happen?**

**E. Do you intend on maintaining your activity (or activities)?**

[If they say no, ask: What makes you say that?]

**F. What do you think could interfere with you continuing your activity (or activities)?**

**C. If you think about knowledge, abilities, skills; what does it take to do X?**

**D. How good would you say you are at X? What makes you say that?**

**E. Do you intend on maintaining your activity (or activities)?**

[If they say no, ask: What makes you say that?]

**F. What do you think could interfere with you continuing your activity (or activities)?**

**D. If you think about knowledge, abilities, skills; how would you describe yourself? How did this change compared to last year?**

**A. What is it like compared to last year? What has changed? What has stayed the same?**
